# Tumor Microenvironment in Adrenocortical Carcinoma: Barrier to Immunotherapy Success?

**DOI:** 10.3390/cancers13081798

**Published:** 2021-04-09

**Authors:** Natalia Georgantzoglou, Stefania Kokkali, Gerasimos Tsourouflis, Stamatios Theocharis

**Affiliations:** 1 First Department of Pathology, Medical School, National and Kapodistrian University of Athens, 115 27 Athens, Greece; natalia.georgantzoglou@gmail.com (N.G.); stefaniakokkali@yahoo.com (S.K.); 2 First Medical Oncology Clinic, Saint-Savvas Anti Cancer Hospital, 115 27 Athens, Greece; 3 Second Department of Propedeutic Surgery, Medical School, National and Kapodistrian University of Athens, 115 27 Athens, Greece; gtsourouflis@med.uoa.gr

**Keywords:** adrenocortical carcinoma, tumor microenvironment, immunotherapy, immune checkpoint inhibitors, PDL-1, survival

## Abstract

**Simple Summary:**

Adrenocortical carcinoma is a rare but aggressive malignancy with poor outcomes even for patients with early-stage disease. Several immunotherapy clinical trials, many of which are still in progress, have yielded modest results so far. In-depth understanding of the tumor microenvironment in other cancer types has helped the scientific community to identify novel therapeutic targets and gain a better insight of cancer biology. The present review aims to provide an update of the current knowledge regarding adrenocortical carcinoma tumor microenvironment and describe how the latter potentially affects patient response to immunotherapy. Following the paradigm of other cancer types, exploiting tumor microenvironment could open new therapeutic avenues.

**Abstract:**

Adrenocortical carcinoma is a rare malignancy with aggressive behavior, with up to 40% of patients presenting with metastases at the time of diagnosis. Both conventional chemotherapeutic regimens and novel immunotherapeutic agents, many of which are currently being tested in ongoing clinical trials, have yielded modest results so far, bringing the need for a deeper understanding of adrenal cancer behavior to the forefront. In the recent years, the tumor microenvironment has emerged as a major determinant of cancer response to immunotherapy and an increasing number of studies on other solid tumors have focused on manipulating the microenvironment in the favor of the host and discovering new potential target molecules. In the present review we aim to explore the characteristics of adrenocortical cancer’s microenvironment, highlighting the mechanisms of immune evasion responsible for the modest immunotherapeutic results, and identify novel potential strategies.

## 1. Introduction

Adrenocortical carcinoma (ACC) is an aggressive malignancy with an annual incidence of 0.5–2 cases per 1 million population. Although it can occur at any age, it has a bimodal distribution, with disease peaks before the age of five and the fifth decade of life, while there is also a predilection for the female gender. Most cases of ACC are considered to be sporadic, however, it can also present as part of hereditary syndromes such as Li–Fraumeni syndrome (LFS), Beckwith–Wiedemann syndrome, Carney complex and Multiple Endocrine Neoplasia(MEN) I [1].

Histologically, malignant cells follow a solid, trabecular, or large nested growth pattern, while tumor necrosis, vascular, and capsular invasion are all commonly present. Weiss score, the most widely used diagnostic system, evaluates the abovementioned features, along with the mitotic rate and nuclear grade, to predict the malignant behavior of adrenocortical neoplasms. Overall, there are three main histologic variants: oncocytic, myxoid, and sarcomatoid, with the oncocytic variant being the most common [1].

Around 50% of the patients present with steroid hormone excess symptoms, and among those, 80% present with Cushing’s syndrome, which is often characterized by remarkably rapid onset. The remaining patients present with tumor growth-related symptoms or are discovered incidentally during imaging studies for unrelated reasons.

Currently, the prognosis for ACC is grim, with the five-year survival rate at 37–47%. For patients with disease at stages I–III, surgical resection is recommended, with adjuvant mitotane (a steroidogenesis inhibitor) therapy in high-risk patients. However, many patients in these stages already have micro-metastases at the time of initial presentation, and therefore surgery is not curative. Approximately one out of two patients treated with surgery will relapse. Adjuvant mitotane treatment has been evaluated in some retrospective studies including heterogeneous populations. It has been demonstrated to improve recurrence-free survival [2], although the results are not consistent across the studies.

For surgically unresectable tumors multi-drug chemotherapy with etoposide, doxorubicin, and cisplatin (EDP), combined with mitotane (M), is currently the mainstay of treatment, while radiofrequency ablation can be employed for local control. EDP-M regimen was evaluated in a prospective phase 2 trial of advanced ACC patients and led to a response rate of 38% and a complete hormonal response in 38% of patients with functional syndrome [3]. The phase 3 First International Randomized Trial in locally advanced and metastatic Adrenocortical Carcinoma Treatment (FIRM-ACT) trial compared first-line treatment with EDP-M versus streptozocin-M. EDP-M was superior in terms of response rate (23.2% versus 9.2%) and median progression-free survival (PFS) (5 versus 2.1 months [4]. Mitotane monotherapy is an option in patients with less aggressive disease. Its use is supported by a German study that reported an objective response rate of 20.5% and a median PFS of 4.1 months [5]). Gemcitabine in combination with capecitabine is usually proposed as a second-line treatment, based on retrospective data lacking a meaningful clinical benefit [6]. Several targeted therapies have also been assessed in small studies with modest results. The poor survival rates, despite the abovementioned therapeutic strategies, highlight the imperative need for novel therapeutic approaches [7].

Immunotherapy (IO) constitutes a new systemic therapy in the armamentarium against cancers, and has resulted in remarkable tumor responses and survival prolongation in various cancers, such as melanoma [8], non-small cell lung cancer [9], renal cell carcinoma [10], and squamous cell carcinoma of the head and neck [11]. In contrast to cytotoxic chemotherapy, which directly “kills” tumor cells, IO induces the activation of the patient’s immune system in order to attack cancer cells, mainly through the activation of cytotoxic T-lymphocytes. The most widely used IO drugs belong to immune checkpoint inhibitors (ICI): PD-1 (programmed cell death protein 1) inhibitors and PD-L1 (programmed death-ligand 1) inhibitors. Both drugs are monoclonal antibodies that inhibit the interaction of PD-1 and PD-L1, and therefore prevent T-lymphocytes’ recognition of cancer antigens through their binding to antigen-presenting cells. Although immunotherapy has undoubtedly revolutionized cancer therapy in the preceding years, results from the ongoing immunotherapy trials for ACC have been modest.

Over the years it has been documented that a major determinant of tumor behavior and responsiveness to immunotherapy is the tumor microenvironment (TME), a dynamic and extremely complex milieu of inflammatory cells, cancer cells, cytokines, extracellular matrix, mesenchymal stem cells, and blood vessels. Cancer cells employ various mechanisms, such as downregulation of HLA molecules and co-stimulatory molecules or apoptosis and the dysfunction of T-cells and dendritic cells, in order to evade immune surveillance and, in fact, they are able to subvert the host’s defense mechanisms into an inflammatory milieu that supports the tumor’s growth and progress. Manipulating TME in an attempt to optimize its immunogenicity has captured the scientific interest as a possible way to modulate responsiveness to immunotherapy [12,13].

In the current review, we aim to explore the characteristics of the immune microenvironment (TIME) of ACC in an attempt to elucidate the possible reasons for immunotherapy resistance and provide an overview of potential new prognostic biomarkers and therapeutic targets.

## 2. Tumor Immune Microenvironment in Adrenocortical Carcinoma

### 2.1. Immune Cells

Tumor-infiltrating lymphocytes (TILs) constitute a polyclonal lymphocyte population that migrates from the bloodstream to the tumor and target tumor associated antigens, playing, therefore, a key role in the anti-tumor immune response. Numerous studies have demonstrated the association of the density and distribution of TILs with patient response to chemotherapy and their overall prognosis in several solid tumors. Denkert et al. demonstrated that TILs are a favorable prognostic factor in HER2-positive breast cancer and triple negative breast cancer [14], while recently, TILs have emerged as a potential biomarker in highly aggressive tumors, such as melanoma [15].

ACC has been traditionally described as an immune deplete tumor, a fact mainly attributed to adrenal glucocorticoid production. In a pan-cancer analysis utilizing mRNA expression of immune-related genes (including PD-L1) in a TCGA (The Cancer Genome Atlas Program) cohort, Pare et al. showed that immune infiltration in ACC is indeed lower compared to other types of cancer [16]. In agreement with this data, an immune genomic analysis of multiple cancer types revealed that ACC, along with uveal melanoma, displayed the lowest leucocyte fraction (calculated based on data of DNA methylation) [17]. TCGA’s integrative genomic analysis of ACC revealed three distinct molecular subtypes (CoC, or cluster of cluster I, II, and III). The expression of immune-related genes is, in general, low, except for the CoC I subgroup, where immune infiltration is observed [18].

However, expression of immune-related genes, such as *ERN1* and *CEP55*, points out the important role of TME in ACC [19]. An increasing number of studies in recent years highlight the significance of TILs as well as other types of immune cells in ACC patients’ prognosis. A recent study using immunofluorescence to quantify infiltrating cell populations in ACC samples, demonstrated that >80% of the samples were infiltrated by lymphocytes with CD8+ cells being the predominant type [20]. It should be noted, however, that the median number of infiltrating immune cells was rather low, which explains the lower quantitative immune gene-related mRNA expression observed in previous studies [16]. Tian et al. applied gene analysis and CIBERSORT algorithms—a computational method for quantifying cell fractions—to a TGCA cohort and showed that the immune infiltrate mainly consisted of T-cells, natural killer cells, mast cells, and macrophages while infiltration levels by each subtype showed strong correlation with the other subtypes. The number of mast cells correlated with survival, as well as with specific changes in signaling pathways [21]. Both the abovementioned studies proved a significant correlation of TIL levels with overall survival (OS) and recurrence-free survival (RFS). Increased levels of TILs were associated with lower pTNM and AJCC staging [21], and notably, metastatic foci seem to be relatively immune deplete compared to primary tumors. This suggests that immune evasion plays a crucial role in disease progression. The association of TILs, and more specifically CD8+ lymphocytes, with prognosis has been demonstrated in pediatric ACC patients as well [22]. Overall, current data suggest that, although lymphocyte infiltration is present in ACC and correlates with patient prognosis, it is quantitatively smaller comparing to other types of cancers.

In addition to the levels of infiltration, the pattern of distribution of infiltrating lymphocytes within the tumor could potentially play a significant role in the dynamics of the TME. The diffuse pattern of CD8+ lymphocytes seems to predominate in ACC compared to the multifocal pattern, which is dominant in benign adenomas [22].

Further investigation regarding the role of immune cells, other than the lymphocytes, in the microenvironment of ACC is warranted. Mast cells constitute a potential target population as they have been positively correlated with prognosis and their presence might exert a regulatory effect on lymph cell populations. Furthermore, emerging data highlight the significance of neutrophils in the microenvironment of many solid tumors, rendering them a potential target in immunotherapy. In a multicenter study, Bagante et al. reported that increased preoperative neutrophil/lymphocyte ratio (NLR) and platelet/lymphocyte ratio (PLR) are associated with decreased post-operative RFS in patients with ACC [23]. Neutrophils are responsible for releasing several chemokines such as TNF-a and VEGF, which are implicated in angiogenesis, steroidogenesis, tumor growth, and metastasis, while, at the same time, interfering with normal T-cell response. It should be noted that the elevated NLR was significantly associated with tumor secretion of glucocorticoids, suggesting that corticosteroid-induced lymphocyte depletion could also be contributing to the observed increased ratio.

### 2.2. Cancer Cells

#### 2.2.1. Altered MHC II Expression

MHC II expression by cortical cells of the innermost zones of the adrenal gland has been well established by previous studies. It is possible that MHC II molecules on cortical cells of zona reticularis play a role in cell-to-cell interactions with androgen producing cells, orchestrating the crosstalk between steroid hormones and infiltrating immune cells. DHEA and DHEA-S exhibit anabolic effects and potentially augment immune response, mitigating, therefore, the effects of locally produced corticosteroids—their exact role, however, remains to be further elucidated, as explained later in this review.

The role of MHC II cortical cell expression in human disease has been well documented. As early as 1988, Jackson et al. described increased expression of MHC II in cortical cells of patients with Addison’s disease [24]. In contrary to autoimmune disease, cancer has been associated with a markedly decreased expression of MHC II in the adrenal gland. Wolkersdorfer et al. reported the absence of MHC II expression on ACC cells, which could result in attenuated interaction of cancer cells with the TCR receptor of T-cells, while at the same time they investigated the interaction of MHC II with Fas/Fas-L in ACC tissue. According to this study, ACC cells exhibit increased expression of Fas-L and decreased expression of Fas receptor [25]. The significance of increased Fas-L expression in immune evasion has been suggested in many different types of cancers, such as melanoma and myeloma, in which Fas-L seems to mediate infiltrating T-cell apoptosis [26]. On the other hand, downregulation of the Fas receptor renders tumor cells resistant to apoptosis, and, according to recent studies, is associated with reduced survival and decreased response to immunotherapy. Xiao et al. reported decreased OS and response to immunotherapy in colon cancer patients with significantly decreased Fas expression [27], while Shibakita et al. demonstrated that Fas expression was an independent negative prognostic factor for RFS in patients with esophageal cancer [28].

In addition, the prognostic role of MHC II has been documented in pediatric ACC as well. Pinto et al. showed that increased MHC II expression correlates with improved prognosis in pediatric patients [29].

#### 2.2.2. TLR4 and CD14 Expression

TLR4 is a trans-membrane protein that belongs to the Toll-like-receptor (TLR) family. TLRs recognize pathogen-associated molecular patterns, with Gram negative derived lipopolysaccharide (LPS) being the most significant among those. Apart from their well-documented role in innate immunity against microbial pathogens, accumulating data highlights the role of TLRs in cancer, which is mostly mediated through receptor stimulation by damage-associated molecular patterns (DAMPs) [30,31]. In order for signal transduction to occur, TLR4 cooperates with co-receptor CD14 and myeloid differentiation factor 2 (MD2), activating ultimately, among others, the NF-κβ pathway.

Depending on the cell type on which TLR4 is expressed, it can either enhance immune response or promote tumorigenesis. More specifically, it has been shown that TLR4 plays a role in promoting the maturation of dendritic cells, thus boosting anti-tumor immunity, while, on the other hand, it stimulates angiogenesis and induces the accumulation of tumor associated macrophages and mesenchymal stem cells, expediting this manner of tumor growth and invasion [32].

Several studies have demonstrated the expression of TLR4 in the normal adrenal cortex and elucidated its role in the adrenal response to sepsis. The significance of TLR4 in adrenal TME, however, had not been widely studied until recently. In a study using immunohistochemical analysis of adrenocortical tumors and real-time PCR in ACC cell lines, Kanczkowski et al. reported a 115-fold decrease in the expression of TLR4 and a 38-fold decrease in the expression of CD14 in ACC tissue when comparing to normal adrenal tissue. The expression of MD2 was reduced as well. In contrast to ACC tissue, adrenocortical adenoma samples seemed to retain, at least partially, TLR4 and CD14 expression [33].

#### 2.2.3. CD276 (B7-H3) Expression

CD276 is a member of the B7 family, with structural similarities to PD-L1 that harbors a dual immunoregulatory role: either co-stimulating APC-induced T-cell activation, or acting as a co-inhibitor of T-cells, contributing therefore to tumor immune evasion. Indeed, accumulating evidence suggests that overexpression of CD276 is implicated in the pathogenesis of many different solid tumors such as lung, prostate, and breast cancer, and has been emerging as a potential immunotherapy target. Apart from suppressing T-cell proliferation and activation, experiments have shown that CD276 downregulates several cytokines, such as IFN-γ, TNF-a, and IL-2, while in vitro studies suggest its potential role in tumor invasion and metastasis as well [34,35].

In a recent study, Liang et al. used immunohistochemistry in 48 ACC samples to assess the overall expression of CD276 in ACC, as well as explore a possible correlation between CD276 expression and patient prognosis. The results of this study showed that >90% of the samples expressed CD276, and more than half of the samples showed moderate to strong expression. Furthermore, higher intensity of CD276 in tumor cells correlated with increased recurrence risk and overall worse prognosis, while at the same time, increased expression of CD276 in tumor vasculature was associated with tumor invasion of adjacent structures and more advanced disease stage [36].

### 2.3. Adipose Stem Cells

Over the previous years, increasing importance has been attributed to the role of adipose stem cells in the TME and their association with increased tumor growth and invasiveness. The correlation of certain malignancies with obesity is well established. The crosstalk between adipose stem cells (ASC) and cancer cells seems to be mediated through the release of several growth factors (VEGF, PDGF, TGF-β), cytokines (IL-6, Il-8, IFN-γ, TNF-a, CXCL2, CXC12), and leptin by the former. Furthermore, several in vitro studies have highlighted the increased expression of matrix metalloproteinases in ASC and cancer cell cocultures, which could be pivotal in a tumor’s potential for migration and invasion [37,38].

Recently, Armignacco et al. performed similar co-culture experiments using human ASC cells and ACC cells (H295R cell line). According to this study, cancer cells showed increased proliferation and invasiveness when cocultured with ASCs in comparison to cancer cell cultures alone. On the other hand, ASCs showed decreased maturation and lipid content in the presence of H295R cells. The chemokine and molecular profile of the cocultures was investigated further, and it was demonstrated that the cocultures showed increased levels of CXC12 and CXC7-chemokines consistently associated with increased tumor migration. In addition, increased levels of leptin and IL-8 seemed to be implicated in the more invasive phenotype of the cancer cells in the coculture, compared to the control [39]. Indeed, several studies in the past have associated leptin with tumor aggressiveness in other solid tumors. Strong et al. reported increased proliferation and metastatic dynamics in ER-positive breast cancer in response to leptin produced from ASCs isolated from obese women [40], while Chen et al. reached similar conclusions regarding proliferation in ovarian cancer cell lines [41].

### 2.4. Immunosuppressive Role of Glucocorticoids on TME

Approximately 60% of ACC patients present with hormonal excess syndromes. Among those, the majority present with either Cushing’s syndrome alone, or a mixed virilization-Cushing’s syndrome, inducing endogenous hypercortisolism. Furthermore, exogenous glucocorticoids are administered to many patients following adrenalectomy or during mitotane treatment. Even in the absence of high serum concentration, intratumoral glucocorticoid concentrations may be high due to activation of steroid synthesis pathway in some tumors [42]. Indeed, CoC II and III molecular subtypes have a steroid-high phenotype and render a worse prognosis, whereas CoC I is characterized by a significant up-regulation of genes in immune-mediated pathways [43].

The various mechanisms by which glucocorticoids impair immunity have been described in detail in numerous studies over the years. In regard to the T-cell response, which has a cardinal role in orchestrating anti-tumor immunity, glucocorticoids deplete T-cells by directly inducing apoptosis, downregulating IL-2 production, and inhibiting their release from lymphoid organs [44]. They also hinder T-cell antitumor function. It is obvious that successful ICI therapy requires intact cytotoxic T-lymphocytes. In addition, high glucocorticoids levels promote tumor cells’ survival and proliferation in vivo [45].

It is of no surprise, therefore, that adrenocortical tumors with glucocorticoid hypersecretion display diminished numbers of TILs, with CD3+ CD4+ being the predominantly affected subset. Landwehr et al. analyzed 146 ACCs and demonstrated a negative correlation between hypercortisolism and overall survival, with immune depleted cortisol-secreting tumors displaying the lowest survival rates (compared to non-immune depleted cortisol-secreting, non-immune depleted non-secreting, and immune depleted non-secreting tumors) [20]. Other studies have demonstrated that glucocorticoids interfere with anti-tumor immune response by downregulating MHCII and TLR-4 [46,47].

In support of the unfavorable glucocorticoid milieu, pembrolizumab administration to a patient with metastatic ACC, Lynch syndrome, and tumor-associated Cushing’s syndrome led rapidly to disease progression (PD) [48]. On the contrary, an impressive response to the drug was reported in another patient with normal cortisol levels, due to concomitant mitotane treatment [49]. Therefore, suppression of glucocorticoids’ negative effect on the immune system seems important to produce meaningful clinical benefit of IO in ACC patients.

Mitotane is effective in controlling glucocorticoid excess by inhibiting glucocorticoid biosynthesis and inducing increased steroid clearance and cortisol-binding globulin, but it takes several weeks [7]. In the case of severe Cushing’s syndrome requiring a rapid control, other agents can be used that inhibit adrenal steroidogenesis. Metyrapone is a steroidogenesis enzyme blocker that can be used concomitantly to chemotherapy and mitotane during the first weeks of treatment until mitotane therapeutic levels are obtained, leading to the rapid resolution of symptoms [50]. Another steroidogenesis inhibitor, which is also administered in patients with Cushing’s syndrome, is ketoconazole. It is less effective than metyrapone though, and it cannot be used in combination with mitotane due to hepatotoxicity. It is also effective in androgen excess. Finally, mifepristone is a synthetic steroid with progesterone receptor antagonist activity and glucocorticoid receptor antagonist activity at higher doses [51] that could also be used. Improvement in 66% of patients during the first month was observed in an international retrospective study [52]. However, there are concerns about mineralocorticoid adverse effects requiring monitoring. Apart from mitotane, all these drugs could be used in combination with IO to overcome hypercortisolism in ACC.

### 2.5. Locally Produced Androgens

The immune modulatory role of androgens is extremely complicated and remains to be fully elucidated. There are reports in the literature that DHEA-S counterbalances the effects of glucocorticoids by interfering with the function of 11β-HSD1, which is responsible for cortisone to cortisol conversion [53]. It has also been suggested that it can potentially augment T-cell response by upregulating Il-2 production, and therefore boost T-cell proliferation, as well as potentiate T-cell mediated cytotoxicity [54]. Canning et al. have also documented a possible positive net effect of DHEA-S on the maturation of dendritic cells [55]. On the other hand, DHEA-S has been reported to downregulate the production of several inflammatory cytokines, such as TNF-a and IL-6 [56].

Further research, therefore, is warranted to clarify the role of DHEA-S in the glucocorticoid-rich microenvironment of adrenal cancer. It should also be noted that pure androgen hypersecreting ACC is extremely rare, as, in most cases, androgen hypersecretion is accompanied by Cushing’s syndrome. Identifying, therefore, the role of androgen hypersecretion on survival could be confounded by the increased cortisol concentrations in the same patients.

### 2.6. Alteration of Oncogenic Pathways

**Figure 1:**
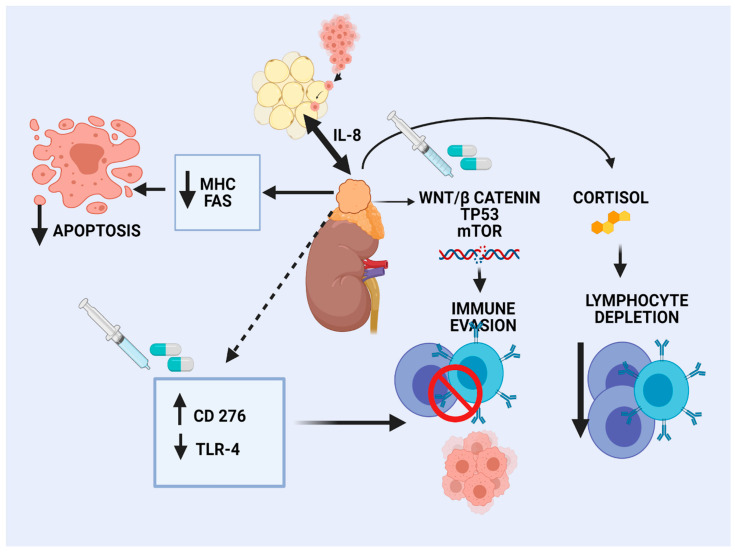
Immune microenvironment in adrenocortical carcinoma.

The landscape of genomic alterations of ACC is complex. Activation of WNT/β-catenin signaling has been recognized as an oncogenic driver in a large subset of ACC patients, mainly in CoC II and CoC III tumors [16]. β-catenin regulates development and homeostasis in different tissues. Preclinical data in melanoma shed light on the effects of WNT/β-catenin pathway activation on tumor immune microenvironment (TIME), characterized by a reduced production of some chemokines, such as CCL4 and subsequent reduced BATF3 dendritic cells. As a result, decreased T-cells recruitment and a defective effector T-cell trafficking into the TME were observed, preventing anti-tumor immunity [57]. Similarly, limited clinical data in human ovarian carcinoma and adenoid cystic carcinomas revealed an association of WNT signaling activation and lack of T-cell infiltration [58]. According to a pan-cancer integrative genomic analysis using the TCGA, activation of WNT/β-catenin signaling was enriched in non-T-cell-inflamed tumors, whereas ACC demonstrated the strongest correlation [59]. In silico analysis of the TCGA ACC tumors revealed that high Catenin β 1B *(CTNNB1)* gene expression correlated with cortisol excess, worsened survival, and decreased immunity, as manifested by fewer TILs [60]. The different elements of the TIME are depicted in Figure 1.

Given the immune exclusion caused by WNT/β-catenin activation, drugs inhibiting the pathway seem rational combination partners for future immune checkpoint inhibitor (ICI) trials. Targeting WNT/ β-catenin signaling is challenging, since it has a critical role in normal tissues homeostasis like bones and various physiological processes. There are no approved drugs in oncology. However, several effectors and inhibitors of WNT/β-catenin pathway have been tested at the preclinical level, as well as in early phase clinical trials of solid tumors and hematologic malignancies [61]. As an example, Porcupine inhibitors act by blocking the secretion of WNT ligands. The frequent deletions or loss of function mutations in the *ZNRF3* gene, a gene which fosters turnover of cell surface Frizzled receptors of WNT, may render ACC harboring these alterations sensitive to these drugs. OMP18RS (vandictumab) is a monoclonal antibody against the Frizzled receptor that has been tested in a phase 1 study of breast pancreatic cancer with promising activity [62], whereas a combination phase 1 study with chemotherapy in pancreatic cancer was terminated due to bone-related adverse events [63]. PRI-724 is a small molecule inhibitor of WNT/β-catenin/CBP (a transcriptional coactivator) signaling, and was evaluated in early phase trials of pancreatic cancer [64].

There are no clinical data on these agents in ACC patients. In vitro studies in ACC models demonstrated that β-catenin targeting inhibited cell proliferation [65,66]. It should be noted that the WNT pathway cross talks with the Notch and Sonic Hedgehog pathways, and thus it is possible that multiple targeting is necessary.

Another important proportion of ACC displays a spectrum of mutations in the p53/Rb pathway, which have been correlated to aggressive histotype and bad prognosis [67,68]. These mutations have also been described as pathogenic in other cancers and represent the most relevant prognostic biomarker in ACC. *TP53* is a tumor suppressor gene encoding for p53 protein, an important regulator of cellular stress response. The majority of *TP53* mutations lead to stabilization and accumulation of the mutant protein, which is protected from ubiquitin-mediated degradation [69]. *TP53* mutations that were identified in ACC result in either a negative function or absence of protein expression. Germline *TP53* mutations are associated with pediatric ACC, as a component of LFS, whereas somatic mutations are observed mainly in adult cases [70].

*TP53* inactivating mutations have been shown to confer an immunosuppressive phenotype, and, due to decreased MHC-I presentation, profound changes in chemokine/cytokine secretion increasing the recruitment and activity of myeloid and Treg cells [71]. Therefore, effector T-cells recruitment in the tumor is suppressed, promoting immune evasion. In the same direction, *TP53* mutations were recently found to downregulate immune-related genes in hepatocellular carcinoma [72]. *TP53* mutations and inactivation have also been reported in cancer-associated fibroblasts (CAF), affecting immune cell composition and function in TIME and leading to pro-inflammatory molecule production [73].

Strategies aiming at restoring p53 function in tumors and CAF could inverse immunosuppressive TME, which is observed in *TP53*-mutated tumors. Pharmacological p53 activation, viral vector-mediated p53 reintroduction, and restoration gene therapy have not provided satisfactory results so far [73]. Given the broad spectrum of *TP53* mutations, mutant-specific reactivating drugs are probably required. Another approach to target p53 is the disruption of the chaperone machinery involved in its impaired degradation.

Additional genetic alterations have been described in different subsets of ACC patients, such as the dysregulation of the mTOR pathway [74]. Substantial evidence supports that the PI3K/AΚΤ/mTOR cascade plays a central role in immune cells’ homeostasis and activation. It can regulate chemokine-mediated immune cells, with diverse roles from promoting T-cell accumulation to immune evasion [75]. The PI3K/AΚΤ/mTOR pathway has been shown to regulate PD-L1 in different cancers, Treg cells, and myeloid-derived suppressor cells infiltration [76]. Therefore, further investigation is required to determine the role of mTOR inhibitors like everolimus and temsirolimus in the immune modulation of selected ACC subgroups. The mTOR pathway has already been suggested as a potential therapeutic target for ACC [77]. Although in vitro testing of these drugs led to the inhibition of ACC cells’ proliferation [78], preliminary clinical data failed to demonstrate efficacy as monotherapy [79].

Combination of IO with drugs targeting the above pathways is a promising strategy to overcome immune exclusion in ACC and warrants extensive research. Furthermore, injection of mature dendritic cells into tumors with β-catenin alterations could also be explored.

## 3. Biomarkers of Immunotherapy in ACC

### 3.1. PD-L1

In recent years, PDL-1 has emerged as the cornerstone of immunotherapy in a wide spectrum of malignancies. PD-L1 aberrant expression by tumor cells exemplifies how cancer often employs host mechanisms to evade the immune response. PD-L1 is a trans-membrane glycoprotein, which, upon binding with its receptor (PD), inhibits T-cell activation and proliferation. PD-L1 expression is upregulated by interferons and normally protects the tissues from collateral damage during inflammatory response against invading pathogens. It also plays a crucial role in self-tolerance and, therefore, disruption in the PD-L1/PD pathway is implicated in autoimmunity. Several types of cancers overexpress PD-L1 and thus evade immune surveillance by T-effector cells [80,81,82].

Existing data signify that only a small subset of ACC tumors expresses PD-L1. Fay et al. examined PD-L1 expression on both cancer and infiltrating immune cells in 28 patients with ACC using immunohistochemistry. Tumor cells expressed PD-L1 in 10.7% of the patients and no significant correlation was found between PD-L1 expression and stage or survival [83]. A retrospective analysis of PD-L1 mRNA in 146 ACC samples revealed heterogeneous expression. High PD-L1 was associated with the presence of a cytotoxic immune response and a longer disease-free survival independently from other prognostic factors [84]. Gene expression analysis demonstrated a correlation of PD-L1 mRNA with an immunological signature, which confers excellent antigen presentation properties and involves the upregulation of T-cell cytotoxicity-related genes. These observations could provide biological plausibility for the positive prognostic value of PD-L1, despite its immunosuppressive role. This is the largest ACC series documenting PD-L1 expression in the literature. In addition, gene expression analysis lacks the inherent immunohistochemistry limitations. However, it should be noted that emerging data from clinical trials in other tumors suggest that PD-L1 expression is not always a reliable predictor of patient response to immunotherapy. Notably, Larkin et al. reported that melanoma patients with PD-L1-negative tumors achieved increased PFS when treated with nivolumab/ipilimumab combination therapy [85]. Nonetheless, identification of other targetable biomarkers in ACC is imperative, in order to identify novel immunotherapy strategies.

### 3.2. TMB

Tumor mutational burden (TMB) is defined as the total number of somatic mutations per coding area of a tumor genome. TMB is a promising emerging biomarker in patient selection for immune checkpoint inhibitor therapy, as an increasing level of evidence suggests that higher TMB is associated with increased responsiveness to immunotherapy [86]. It is hypothesized that the increased load of neoantigens, associated with higher TMB, harbors immunogenic properties that trigger the host’s immune anti-tumor response.

TMB varies not only between different cancer types but also between distinct histologic variants. Current research data suggest that ACC harbors an intermediate TMB [87], which appears to be higher in myxoid and conventional subtypes and lower in the oncocytic subtype [68]. It has also been demonstrated that metastatic foci display a higher TMB compared to primary tumors [88]. According to Bonneville et al., TMB correlates with microsatellite instability (MSI), as the MSI-high ACC samples in the study had a significantly higher number of somatic mutations compared to microsatellite-stable (MSS) ACC samples [89]. In accordance with this data, there have been reports of ACC patients with high mutational load that harbored MSH2 mutations and demonstrated long-term response to immunotherapeutic agents [90].

### 3.3. MSI/MMR

Microsatellites are short repetitive DNA sequences consisting of 1–6 bp motifs, found in both coding and non-coding areas of the human genome. Their repetitive nature renders them susceptible to duplications or deletions that are normally repaired by the mismatch repair system (MMR). Defects in MMR, stemming from germline or somatic mutations, lead to the inability of error correction during replication and the generation of new (mutant) alleles, a condition known as MSI. Germline mutations in MMR are responsible for Lynch syndrome, which has traditionally been associated with colon, ovarian, and endometrial cancer, but the spectrum of associated cancers is much wider than originally thought.

An increasing number of studies have linked the presence of defective MMR (dMMR) with responsiveness to immunotherapy [91,92], which can be attributed to the immunogenic properties of the neoantigen load [93]. Notably, in 2018, the FDA approved the use of pembrolizumab for adult and pediatric patients with defective MMR (MSI-high) solid tumors of all types.

Accumulating evidence over the preceding years suggests that ACC could be a Lynch syndrome-associated cancer. There have been several reports of patients with known Lynch syndrome, positive for germline MSH mutations, presenting with ACC [48,94,95]. In addition, a case of ACC as the only manifestation of Lynch syndrome, in a patient with family history of Lynch-associated cancers, has also been reported [96]. MSH2 mutation is the most common mutation reported among the existing cases of Lynch-associated ACC in the literature. In a recent prospective study evaluating patients with known ACC for MSH mutations, the prevalence of Lynch syndrome among ACC patients was estimated at 3.2%, which is significantly higher than the estimated percentage in the general population [97]. Apart from the loss of MSH2 expression, which had already been reported, immunohistochemical staining revealed loss of MSH6 and MLH1 expression as well in two out of four and one out of four tumors, respectively. MSI analysis revealed that all four tumors were MSS. In contrary, in a pan-cancer analysis by Bonneville et al., MSI was detected in 4.3% of ACCs, which is lower than the estimated percentage in endometrial and colon carcinoma, but higher than the average value for the 39 cancer types examined in the study [89].

Overall, based on currently available research data, patients newly diagnosed with ACC could potentially benefit from genetic testing, especially in cases with positive personal or family history of other Lynch-associated cancers. Since ACC is a rare malignancy, further research on larger samples is warranted in order to reach safe conclusions regarding MSI and its potential as a biomarker for immunotherapy selection.

### 3.4. Other Biomarkers

Melanoma antigen recognized by T-cells (MART1/Melan-A) is among the first characterized tumor antigens in melanoma cells. Immunoreactivity to MART1/Melan-Amelan-A/MART1, which is expressed by melanoma cells, has also been described in both primary and metastatic ACC [98,99]. The immunogenic antigen is recognized by cytotoxic T-cells. This non-mutated overexpressed antigen has been traditionally used in immunotherapy clinical trials of melanoma patients, testing dendritic cells-based vaccines leading to increased frequencies of circulating MART-1 specific CD8+ and CD4+ T-cells [100]. In this direction, cancer vaccines or adoptive cell transfer employing MART-1 are interesting immunotherapeutic strategies that could be explored in patients with immunologically non-silent tumors.

## 4. Immunotherapy Trials in ACC

### 4.1. Clinical Trials with Immune Checkpoint Inhibitors

Inspired by the results from other cancers, a number of patients with advanced ACC have been treated with the PD-1 inhibitors pembrolizumab and nivolumab. Pembrolizumab, in particular, was tested in three phase 2 single-arm trials with modest results. The first one included a cohort of 16 pretreated ACC patients, among patients with various rare malignancies [101]. Pembrolizumab monotherapy resulted in two PR and two disease stabilizations. In the second one, nine out of 39 patients achieved PR and seven stable disease (SD), with a median PFS of 2.1 months and OS of 24.9 months [102]. No correlation with known IO biomarkers was found in the above two trials. Furthermore, four ACC pediatric patients were included in an international study of PD-L1-positive solid tumors [103], of whom two achieved a PR. In a case-series of six ACC patients treated with pembrolizumab in combination with mitotane, two PR and four SD were noted [104]. A limited number of case-reports on pembrolizumab alone or in combination with mitotane in advanced ACC have also been published [48,49,90]. Interestingly, a remarkable response was noted in two patients with MSI, one of whom also had a high TMB [49,90].

Nivolumab monotherapy was evaluated in a small multicenter phase 2 trial with 10 advanced ACC patients in the USA [105]. Despite the low median PFS of 1.8 months, two patients achieved SD and one PR. It should be noted that the patient who responded had a highly positive tumor for PD-1 (>50%).

Finally, the efficacy and safety of the novel PD-L1 inhibitor avelumab was assessed in the phase 1b study JAVELIN, consisting of consecutive parallel group expansion cohorts in selected tumor types. The ACC cohort included 50 pretreated patients with metastatic disease and represents the largest prospective immunotherapy trial in ACC [106]. In conjunction with the results of PD-1 inhibitors, avelumab administered either as monotherapy or in combination with mitotane failed to demonstrate substantial activity, resulting in three PR and 21 SD and a median PFS of 2.6 months. Currently available results from ACC immunotherapy clinical trials are summarized in Table 1.

Taken together, the results of the above trials suggest that immune checkpoint blockade in ACC results in relatively low response rates and poor PFS. A factor that should be taken into consideration is the effect of previous treatments. The vast majority of patients who participated in these trials were pretreated with at least one line of chemotherapy and mitotane, and some of them were heavily pretreated. In the JAVELIN trial, the number of prior lines of therapy correlated inversely with response rate to avelumab. Analysis of the responders to pembrolizumab in the largest of the two phase 2 trials revealed that all of them were pretreated (versus 72% of the whole population), but most of them had received mitotane and only 33% of them cisplatin-based chemotherapy [102]. A high number of prior therapies might signify advanced disease with increased tumor burden and number of resistant cancer cells. Multiple chemotherapies can also cause immunosuppression.

In addition, genetic aberrations of ACC patients could be associated with response to ICI. The small number of patients in most of these trials renders the analysis of their molecular profile extremely difficult. In the nivolumab study, NGS was realized in five out of ten patients, revealing a p53 abnormality [105]. In the phase 2 study with pembrolizumab, NGS was performed on all tumors, and different genetic alterations were pointed out [102]. No correlation with efficacy data could be established in any of these studies. However, it would be interesting to analyze the molecular abnormalities in responders and non-responders to ICI, to better select patients for future trials.

In view of the modest results of ICI monotherapy, combination trials of these drugs are currently ongoing. Two phase 2 trials are evaluating the combination of nivolumab with the CTLA-4 inhibitor ipilimumab in rare metastatic tumors and genitourinary malignancies, respectively, including ACC (NCT02834013, NCT03333616). In addition, a phase 1/2 research study has been designed to explore the role of stereotactic radiation therapy given concomitantly or consecutively to ipilimumab in advanced solid tumors (NCT02239900). An international phase 1/2 trial investigating the combination of nivolumab with EO2401, an innovative cancer peptide therapeutic vaccine based on the homologies between tumor-associated antigens and microbiome-derived peptides, is currently recruiting ACC patients (NCT04187404). Finally, a phase 2 study will evaluate the efficacy of the anti-PD1 agent camrelizumab combined with the tyrosine kinase inhibitor apatinib in advanced ACC (NCT04318730).

### 4.2. Other Immunotherapeutic Approaches

Early attempts of immunotherapy in ACC were based on an autologous dendritic cell vaccination. Autologous dendritic cells were pulsed with tumor lysate, which was used as antigen source [107]. Despite the induction of specific Th1 immunity, no impact on the clinical outcome of two vaccinated patients with metastatic disease was noted. This can be the basis for future vaccination trials.

The expression of IL-13-Ra2 by some tumors provided a scientific rationale for a phase 1 study of the recombinant cytotoxin IL-13-PE, consisting of human IL-13 and a truncated form of Pseudomonas exotoxin (PE) A in ACC patients [108]. One SD was observed among the five patients who were treated with the maximum-tolerated dose, whereas the rest had progressive disease. All patients developed neutralizing antibodies against PE. The patient with SD had the highest expression of IL-13-Ra2 and the latest development of neutralizing antibodies.

Strategies exploiting specific tumor antigens, such as steroidogenic acute regulatory (StAR) protein, also seem promising. A transgenic mouse model expressing StAR protein, as most human ACC, was constructed and immunized using DNA plasmids encoding StAR and a recombinant vaccinia virus vector [109]. A specific immune response was observed, breaking tolerance towards StAR and paving the way to similar approaches in human ACC.

Another target with great translational potential is CD276 (or B7-H3), a checkpoint molecule expressed in most cancer cells and tumor-associated vascular cells in ACC. Its expression has been associated with a bad prognosis [40]. CD276 targeted CAR T-cells were shown to have anti-tumor activity in pediatric solid tumor xenografts, depending on surface target density [110]. This novel therapy could be assessed in ACC patients.

## 5. Conclusions

Over the preceding years, TME has been recognized as a key contributor to tumor behavior, regulating disease progression and response to treatment [111,112,113]. The TME of ACC has not been extensively studied until now, and detailed data on its signaling cascades, cell to cell interactions, and the interplay between corticosteroids and local cell populations is limited. Overall, existing literature suggests that relative corticosteroid-induced immune cell depletion (comparing to other tumors), along with low load of immunogenic molecules, such as MHC II and TLR-4, and a high load of T-suppressive CD 276 interfere with the host’s anti-tumor response. Additionally, consistently low PDL1 expression and activation of oncogenic pathways, leading to the impairment of CD8+ production, add to the complexity of the system and altogether explain the modest immunotherapeutic results that have been obtained so far.

Future research endeavors could focus on manipulating TME by targeting key immune-related molecules, such as CD 276, which is an emerging target in the immunotherapeutic approach of other solid tumors, and is apparently overexpressed in ACC, Further research in order to unravel the exact role that corticosteroids and androgens play in orchestrating tumor-immune interactions in TME could also prove to be of paramount importance in developing novel therapeutic strategies. The relatively low levels of lymphocyte infiltration observed in ACC TME compared to other tumors is another challenge that should be addressed, as T-cell infiltration is necessary for immunotherapeutic efficacy. Following the treatment paradigm in other cancers, combination studies of immunotherapy with chemotherapy or radiation in order to render “cold” tumors “hot’’ (immunogenic) would be clinically relevant in ACC as well. Additionally, drugs targeting p53 and WNT/b-catenin signaling, which are in early clinical development, could also be tested in ACC in combination with immunotherapy, with special consideration to tissue selectivity which is an inherent problem of these drugs.

Another key aspect that warrants further research is the association of ACC with Lynch syndrome. The incidence of ACC in patients with known MSH germline mutations or other Lynch-related cancers should be assessed in sufficiently large patient cohorts in order to determine whether genetic testing is indicated in every patient presenting with ACC. This could prove of particular clinical interest as—despite the overall modest immunotherapy results in the metastatic setting—there are case reports of improved response in patients with documented MSI.

Overall, TME in ACC and its correlation with immunotherapy efficacy has been relatively understudied until now. Although the field of immunotherapy is promising and always evolving, future studies have to fill the knowledge gaps that hinder a better understanding of the tumor’s behavior and its potential response to novel therapeutic approaches.

## Figures and Tables

**Table 1:** Immunotherapy trials in ACC.

Drug	Target	Number of Patients	Type of Study	Results	Reference
Pembrolizumab	PD-1	16	Clinical Trial (Phase II)	PR in 12.5% of patients SD in 12.5% of patients	[101]
		39	Clinical Trial (Phase II)	PR in 23% of patients SD in 17.9% of patients PSF = 2.1 mo OS = 24.9	[102]
		4 (pediatric)	Clinical Trial (Phase II)	PR in two patients	[103]
Pembrolizumab + Mitotane	PD-1	6	Case Series	PR in two patients SD in four patients	[104]
Nivolumab	PD-1	10	Clinical Trial (Phase II)	PR in one patient SD in two patients PSF = 1.8 mo	[105]
Avelumab	PD-L1	50	Clinical Trial	PR in 6% of patients SD in 42% of patients PSF = 2.6 mo	[106]
Nivolumab + Ipilimumab	PD-1/CTLA-4	ongoing	Clinical Trial	ongoing	

PR, partial response; SD, stable disease; PFS; progression free survival; OS, overall survival.

## Data Availability

Not applicable.

## Abbreviations

ACC	adrenocortical carcinoma
APC	antigen presenting cells
ASC	adipose stem cells
CAF	cancer associated fibroblasts
CoC I	cluster of cluster I
DAMPs	damage associated molecular patterns
DHEA	Dehydroepiandrosterone
DHEA-S	dehydroepiandrosterone sulfate
dMMR	defective mismatch repair
ICI	immune checkpoint inhibitors
IO	Immunotherapy
LFS	Li Fraumeni syndrome
LPS	Lipopolysaccharide
MART-1	melanoma antigen recognized by T-cells
MD2	myeloid differentiation factor 2
MSI	microsatellite instability
MSS	microsatellite stable
NF-κΒ	nuclear factor κ-light-chain-enhancer of activated B cells
OS	overall survival
PD	progressive disease
PD-1	programmed cell death protein-1
PDL-1	programmed cell death ligand-1
PFS	progression free survival
RFS	recurrence-free survival
SD	stable disease
StAR	steroidogenic acute regulatory protein
TCR	T-cell receptor
TIME	tumor immune microenvironment
TILs	tumor infiltrating lymphocytes
TLR	Toll-like receptor
TMB	tumor mutation burden
TME	tumor microenvironment
TNF-α	tumor necrosis factor-α
Treg	T regulatory cells
VEGF	vascular endothelial growth factor
